# Supplementary material for: White Collar 1 Modulates Oxidative Sensitivity and Virulence by Regulating the HOG1 Pathway in Fusarium asiaticum
Source: Microbiol Spectr. 2023 May 17;11(3):e05206-22. doi: 10.1128/spectrum.05206-22 (PMC10269464; doi:10.1128/spectrum.05206-22)
Supplement: Supplemental file 1 — Supplemental material. Download spectrum.05206-22-s0001.pdf, PDF file, 0.2 MB [file spectrum.05206-22-s0001.pdf]

Supplementary Materials

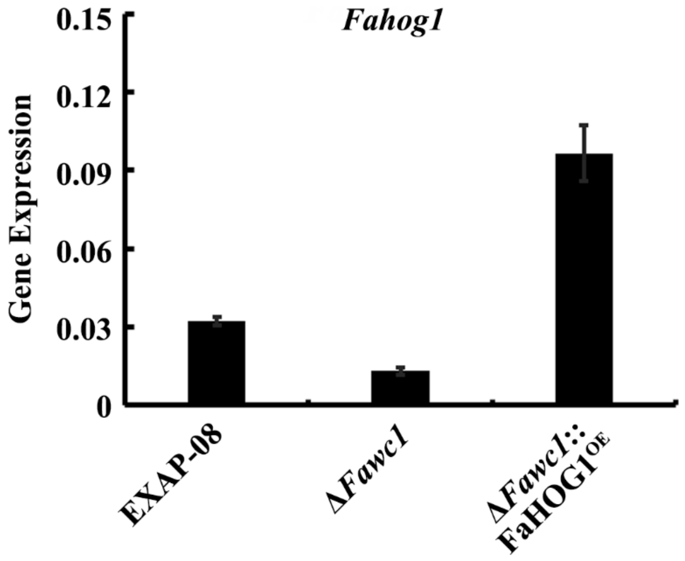

Figure S1. Expression levels of Fahog1 in WT, Δ*Fawc1*, and Δ*Fawc1*::FaHOG1^{OE} transgenic strains.

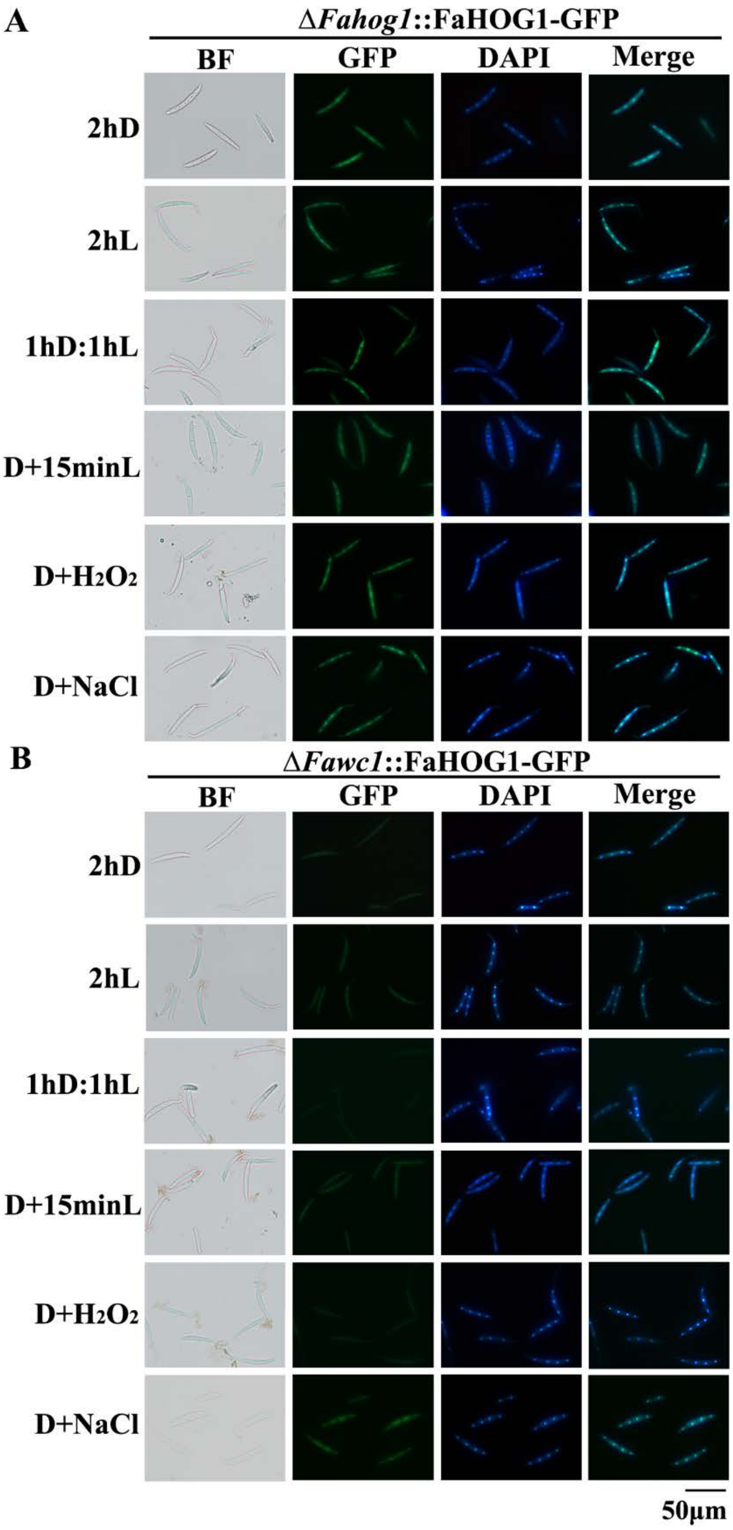

Figure S2. Microscopic views showing more spores with expression and subcellular localization patterns of FaHOG1-GFP. **(A)** The expressed FaHOG1-GFP signals were accumulated in nuclei upon light, oxidative, and salt stress stimuli in the Δ*Fahog1*::FaHOG1-GFP strain. **(B)** Expressing and nucleus localization of FaHOG1 could induced by osmotic, but not by light and oxidative stimuli in the Δ*Fawc1*::FaHOG1-GFP strain.

Supplementary Table S1 Oligonucleotide primers used for target amplification in this study

Primer	Nucleotide sequence (5‘-3’)	Description
F (β-*tubulin*-Q)	ACGGCACGGGGAACATACTTG	Primers to amplify of the *Fatublin* gene in quantitative real-time PCR
R (β-*tubulin*-Q)	TCGAGCGCATGAGCGTTTACT	
F (*Fahog1*-Q)	GCTCCATCATTTGTTCGCAT	Primers to amplify of the *Fahog1* gene in quantitative real-time PCR
R (*Fahog1*-Q)	GTTCCTCGACACCGTCTC	
F (*Fahog1*-GFP)	GATTACGCCGAATTCGAGCTCCATCGTATGCTCTCTATCATGG	Primers to amplify fragment for construction of *Fahog1* complementation vector
R (*Fahog1*-GFP)	GAAACCATCCCGGGTTTCTCGAGTTGTCCATTAAACTGCTC	
F (*hyg*)	CGGCGTAGGGTTGTTCC	Primers to identify resistance gene in deletion mutant
R (*hyg*)	TGGCGACCTCGTATTGG	
F (*FaAtf1*-Q)	GGACATGGACATGATGTCTG	Primers to amplify of the *FaAtf1* gene in quantitative real-time PCR
R (FaAtf1-Q)	CGTTCTCAGTGCTGAACATC	
F (*FaSod*-Q)	GGAGTCTGAGTCTGCTCCC	Primers to amplify of the *FaSod* gene in quantitative real-time PCR
R (*FaSod*-Q)	GGCATTGCCCTGGCCGTCA	
F (*FaCAT*-Q)	GGCAGCGAGGTATACCTGCT	Primers to amplify of the *FaCat* gene in quantitative real-time PCR
R (*FaCAT*-Q)	CTCAATAGCGTTGAAGAGGTC	
F (*FaPbs2*-Q)	TTCAGAGTGACGTCTGGAGTC	Primers to amplify of the *FaPbs2* gene in quantitative real-time PCR
R (*FaPbs2*-Q)	TCGATGAGCCAGGGATGCTTC	
F (*FaSsk2*-Q)	CGAGGACGAGCAAGTCATCA	Primers to amplify of the *FaSsk2* gene in quantitative real-time PCR
R (*FaSsk2*-Q)	CCTGTCATGGATCGGTTCGG	
F (*FaSsk1*-Q)	GACCGCCTGGATAACACTTC	Primers to amplify of the *FaSsk1* gene in quantitative real-time PCR
R (*FaSsk1*-Q)	CGACTTTGCCTTGGCTGC	
F (5’-*Fahog1*)	CATCGTATGCTCTCTATCATGG	Primers to amplify upstream fragment for

R (5'-*Fahog1*)	CCACAGCTGCAGTCTAGAGCGGTGAATATGTGGTTGTTGAG	generating *Fahog1* deletion mutant
F (*Fahog1-hyg*)	CTCAACAACCACATATTCACCGCTCTAGACTGCAGCTGTGG	Primers to amplify resistance fragment for
R (*hyg-Fahog1*)	CTTTATTAACCACTTCTCCCTTCGGGATCCGCTTAGACAAC	generating *Fahog1* deletion mutant
F (3'-*Fahog1*)	GTTGTCTAAGCGGATCCCGAAGGGAGAAGTGGTTAATAAAG	Primers to amplify downstream fragment for
R (3'-*Fahog1*)	CATGGACTATGAGACTGACC	generating *Fahog1* deletion mutant
F (*Fahog1*)	TCAGAACCTCGACTTTCCGCAT	Primers to amplify of *Fahog1* sequence for
R (*Fahog1*)	CCGTCTCCGTGAGAGTCTTAC	clone and sequence
F (*Fahog1*-OE)	GTAGATCCTCTAGGTACCCGGGGATCCATGGCCGAGTTTGTAC GCGC	Primers to amplify fragment for generating *Fahog1* overexpression mutant
R (*Fahog1*-OE)	GAGGCCATCTCGAGCGGGATCCTTGTCCATTAAACTGCT	
F (*FaCat4*-Q)	GGAAGTTATGGCCGCACCC	Primers used for amplification of the *FaCat4*
R (*FaCat4*-Q)	GAACATGCGTGCTTGGAGG	gene in quantitative real-time PCR
F (*FaCat6*-Q)	CGACGTCCGTTACTCCAACG	Primers used for amplification of the *FaCat6*
R (*FaCat6*-Q)	CTTCGTCGTTCATGGCCAT	gene in quantitative real-time PCR
F(*Fawc1*-GFP)	GATTACGCCGAATTCGAGCTCGCCACGGACTAGGTACTGAAG	Primers to amplify fragment for construction
R(*Fawc1*-GFP)	GAAACCATCCCGGGTTTCTCGAGAGATTGGCTTGTCTCGCG	*Fawc1*-GFP complementation vector
F(PNR2)	GATAAGTCGTGTCTTACCGG	Primers to identify construction in PNR2
R(PNR2)	GGACAAGACCGAATCAATGC	vector

Supplementary Table S2 Oligonucleotide probes used for EMSA assay

Probe Names	Sequences
Probe 1 (Forward)	5' – CAACGCCTTGATACGACCAAAAGGAAAAGACGGTCACAAAAAAAAGTC – 3'
Probe 1 (Reverse)	5' – GACTTTTTTTTGTGACCGTCTTTTCCTTTTGGTCGTATCAAGGCGTTG – 3'
Probe 2 (Forward)	5' – CGGTCGCCAACGCCTCTTCCCGCCCCGCGCTCAATACGCTATACTCATCGACATC – 3'
Probe 2 (Reverse)	5' – GATGTCGATGAGTATAGCGTATTGAGCGCGGGGCGGGAAGAGGCGTTGGCGACCGG – 3'
Probe 3 (Forward)	5' – TCGCTTTTTTTTACTGCTACCCCGGTCACGGTCATCGTCTTTTTCTCGATCGTCGATTTC – 3'
Probe 3 (Reverse)	5' – GAAATCGACGATCGAGAAAAAGACGATGACCGTGACCGGGGTAGCAGTAAAAAAAAGCGA – 3'
Probe 4 (Forward)	5' – CCATCCCATTTCCATCCCCAGCAACCCATCCAATCCAACAACTAGATTGG – 3'
Probe 4 (Reverse)	5' – CCAATCTAGTTGTTGGATTGGATGGGTTGCTGGGGATGGAAATGGGATGG – 3'
Probe 5 (Forward)	5' – CCGCCATCCATCATCTATACCTCATCTTTGTGTCCTGTTCCTCGTTCTG – 3'
Probe 5 (Reverse)	5' – CAGAACGAGGAACAGGACACAAAGATGAGGTATAGATGATGGATGGCGG – 3'
Probe 6 (Forward)	5' – GGAAACTCTTCACCTTTCACTTTCACTTTCACTTTCAACCTCAAA – 3'

Probe 6 (Reverse)	5' - TTTGAGGTTGAAAGTGAAAGTGAAAGTGAAAGGTGAAGAGTTTCC - 3'
Probe 7 (Forward)	5' - TTGCCACTCCTCCATGCGATCCATCTCGATCTACATGTAA - 3'
Probe 7 (Reverse)	5' - TTACATGTAGATCGAGATGGATCGCATGGAGGAGTGGCAA - 3'
Probe 8 (Forward)	5' - CCAGCACGATAGCTTCTAATGCTGGCCTTGTGCGATCCATGTGC - 3'
Probe 8 (Reverse)	5' - GCACATGGATCGCACAAGGCCAGCATTAGAAGCTATCGTGCTGG - 3'
